# Biographical Sketch of the Late Dr. Rush

**Published:** 1875-04-15

**Authors:** 


### BIOGRAPHICAL SKETCH OF THE LATE DR. RUSH.

*Philadelphia Correspondence of Boston Medical and Surgical Journal, April 1st, 1875.*

A MOST interesting biographical sketch of the life of the late Dr. Rush, whose brilliant success in medicine will not soon be forgotten, was recently published in one of the daily journals. It was he who said, "Let my epitaph be, 'He fed fevers.'" For such was his clear-sightedness that, even in the day of starvation diet, he saw how much wiser it was to supply daily fuel to the fires of fever than to allow it to devour the body of his patient. He made enemies by this as well as by other independent and original modes of treating disease. I think a brief *resume* of this sketch will interest your readers.

Born in a village just out of Philadelphia, in 1745, Rush received his degree of A. B. at Princeton College in 1760, and, although a mere boy, was the peer of any of his fellows in all his collegiate studies. Immediately after completing his collegiate course, he entered upon the study of medicine, and was one of the ten students who attended Dr. Shippen's course of anatomical lectures, the first ever given in this country. Gifted with an investigating mind, Rush studied laboriously until 1766, when he sailed for Edinburgh, and, after two years of hard work, received his degree of M. D. from the university of that city. He next gave a year to continental travel, mingling with the most cultivated medical men in Paris and London. At the close of this year he returned to Philadelphia, and began the practice of medicine. At the very early age of twenty-four, he was elected Professor of Chemistry in the College of Philadelphia, and became a frequent contributor to medical and general literature. The present University of Philadelphia was at this time in process of development, lectures having been delivered at irregular intervals for a year. The acquisition of young Rush completed the faculty of the first medical school in the United States. Some fifteen years afterward, the primitive institution was merged into the present university, and Dr. Rush, recognized as one of the most brilliant young physicians of the country, held the position of professor of the institutes of medicine, and of practice, as well as of clinical medicine. He was an exceedingly minute man, garnering with care every floating fact, theory and incident for future use, absorbing everything, forgetting nothing. In 1790, after a practical experience of twenty years, he published his new principles of medicine. His views met with strong opposition. He had great confidence in the lancet, and a lasting faith in the power and utility of calomel, which he termed "the Samson of the materia medica." His opponents accepted the illustration, jocosely remarking, "It has slain its thousands." These powerful agencies, under the skillful control of Dr. Rush, were productive of beneficial results.

In 1793, Philadelphia was terribly stricken by yellow fever. It raged from July to November, creating an average death-rate of forty daily, with a final aggregate of five thousand victims. The city was panic-stricken. During this frightful crisis, Dr. Rush made herculean efforts to subdue the [illegible]my, working practically a portion of his time, and devoting the remainder to a thorough scientific analysis of the disease. He visited one hundred and fifty patients weekly, and saved thousands of lives by his original and judicious treatment. His special method of handling the fever, in spite of its success, was bitterly reviled. Journalists, pamphleteers, and scurrilous anonymous writers attacked him with fierce malignity, until the discussion, primarily based on questions of professional skill, sank into petty persecution. He was stigmatized as a murderer, and threatened with expulsion from the city. As Harvey injured his professional prospects by his theories concerning the circulation, and was hooted as a common fool, so did Dr. Rush lose public confidence because he bravely turned his back upon the beaten path to subdue a pestilence heretofore never vanquished. But he moved on, calmly and unfalteringly.

On the termination of the fever, a motion to thank the faculty in general, and Dr. Rush in particular, for eminent services during the epidemic, was offered at a large public meeting, but not a man in the audience was bold enough to second it, and it failed.

Dr. Rush finally turned upon one pamphleteer of bitter force and invective, who violently attacked him, and made him pay five thousand dollars for his sport. This was one of many assaults made upon Dr. Rush, but he survived them all, and built up and retained the largest practice in Philadelphia. A few years afterwards a reaction set in, soon after the gift of a gold medal which Rush received in 1805 from the King of Prussia, in recognition of his replies to certain questions touching the treatment of yellow fever. For the same reason the Emperor of Russia sent him a brilliant diamond ring, and the Queen of Etruria a gold medal.

Dr. Rush was a voluminous writer. Out of seven large volumes six were upon medical subjects. His *Medical Inquiries and Observations*, *Diseases of the Mind*, *Medical Tracts*, *Health, Temperance and Exercise*, gave him a high reputation at home and honorable notice abroad. In early life he found time to study politics, not as a selfish partisan, but as an honest, noble-minded citizen. In 1776 he was a member of the celebrated Congress which signed the Declaration of Independence. In 1777 he was appointed physician-general of the military hospitals of the middle department. In 1787 he was a member of the Pennsylvania convention which ratified the Federal constitution. In 1799 President Adams appointed him treasurer of the United States Mint, solely on account of his faultless character and sterling integrity. The office was an unsolicited gift, and he held it fourteen years.

But few cities in Europe, and certainly none in this country, have such a variety of charitable institutions as Philadelphia. No one citizen contributed more to the organization of many of these than did Dr. Rush. In 1785 he planned and organized the Philadelphia Dispensary, the first institution of the kind in America. He was president of the Philadelphia Medical Society; president of the Philadelphia Society for the Abolition of Slavery; founder of the Philadelphia Bible Society; vice-president of the celebrated American Philosophical Society; a strong, practical friend of the temperance cause, and his work, *An Inquiry into the Effects of Ardent Spirits upon the Human Body and Mind*, is full of valuable information. He was a lofty-minded Christian gentleman. His private life was one of unsullied purity, and his public career unsurpassed in its many brilliant developments and practical results for the common good of his country and his fellow-men.

In my next letter I hope to give you some interesting facts concerning Dr. Keen's later experiences in the use of chloral as a preservative agent.

PHILADELPHIA, March 23, 1875.

